# Influence of Judo Experience on Neuroelectric Activity During a Selective Attention Task

**DOI:** 10.3389/fpsyg.2019.02838

**Published:** 2020-01-09

**Authors:** Heloiana Karoliny Campos Faro, Daniel Gomes da Silva Machado, Henrique Bortolotti, Paulo Henrique Duarte do Nascimento, Renan Cipriano Moioli, Hassan Mohamed Elsangedy, Eduardo Bodnariuc Fontes

**Affiliations:** ^1^Department of Physical Education, NEUROex – Research Group in Physical Activity, Cognition and Behavior, Federal University of Rio Grande do Norte, Natal, Brazil; ^2^Graduate Program in Collective Health, Federal University of Rio Grande do Norte, Natal, Brazil; ^3^Graduate Program in Neuroengineering, Edmond and Lily Safra International Institute of Neuroscience, Santos Dumont Institute, Macaíba, Brazil; ^4^Graduate Program in Bioinformatics, Digital Metropolis Institute, Federal University of Rio Grande do Norte, Natal, Brazil

**Keywords:** electroencephalogram, event-related potentials, event-related spectral potential, brain oscillations, sport expertise, executive function, attention-inhibition

## Abstract

**Objective:**

We compared the cognitive performance and neuroelectric responses during a selective attentional task in judo athletes with different levels of expertise.

**Methods:**

Judo black and white belt athletes performed both general and specific fitness tests while simultaneously completing a Stroop color-word test recorded by 64 electroencephalogram channels.

**Results:**

Cognitive behavioral performance and event-related spectral perturbation (ERSP) present no differences between groups. However, the topographic analysis found different neural source patterns in each group. Judo black belts compared to judo white belts presented a greater peak amplitude of P300 in the middle frontal gyrus and of N200 in the cuneus, but slower latency of P300 in the precuneus.

**Conclusion:**

Despite no difference in cognitive behavioral performance, judo expertise causes a difference in the allocation of attentional and conflict detection neural resources.

## Introduction

In martial arts combats, athletes are exposed to a high cognitive load; this includes sticking with a strategy, reading the opponent’s actions, planning to attack, listening to the coach, ignoring the crowd, and weighing combat status (i.e., score, time and rounds left, etc.). Most of these demands relate to the executive function, computational processes, involved in the selection, scheduling, and coordination of complex cognitive functions (Hillman et al., 2008). In general, executive function is categorized into three core aspects- inhibitory capacity (interference control, selective attention, cognitive inhibition, response inhibition or self-control: resisting temptations and resisting acting impulsively), working memory, and cognitive flexibility (quickly and flexibly adapting to changed circumstances) (Diamond, 2013). In an athletic context, especially in combat modalities, selective attention (i.e., attending to relevant and ignoring irrelevant stimuli) seems to play a key role in the cognitive demand of sports (Sánchez-López et al., 2013). Given the limited human capacity of processing information at a given time, greater abilities for selective attention helps athletes decide which stimuli should be attended to (relevant; e.g., coach instruction) and/or which stimuli should be ignored (irrelevant; e.g., crowd shouting) (Diamond, 2013). Besides selective attention, decision making (e.g., when and how to attack or defend) is considered the main neuropsychological element given the unpredictability of combats (Supiński et al., 2014; Fortes et al., 2017).

In this regard, a meta-analysis showed that athletes demonstrate quicker and more accurate responses in sports-specific tasks when compared to novices, which is likely linked to the specialization of cognitive and attentional factors (Mann et al., 2007). From a neural perspective, the need to constantly evaluate and adjust movements according to the constraints requires athletes to dedicate large amounts of cognitive resources (Yarrow et al., 2009). In fact, beyond physical and technical performance, a recent systematic review showed that martial art practice through the life span also results in improvements in different aspects of cognitive performance (Zou et al., 2018). Despite this, it has been suggested that less brain activation is an adaptive response to training (Del Percio et al., 2008; Callan and Naito, 2014).

The Stroop color-word test (SCWT) has been widely used to measure selective attention and inhibitory control, which is based on the fact that individuals often take longer to name a color of a color-word printed in a different ink color (i.e., incongruent stimulus with a semantic conflict), such as naming the word GREEN in blue ink than when color-word and ink color are matched (i.e., congruent stimuli; the word RED in red ink) (Diamond, 2013). This specific feature is called “Stroop effect/interference.” In the matching version of SCWT, a color-bar and a color-word must be compared. The meaning of the word must match to the colored-bar while the color of the word represents an irrelevant stimulus feature (or dimension) that should be ignored. If the word meaning and the color of the bar match (i.e., are congruent; indicate the same color meaning), individuals issue a go response (simple button press), but if the word meaning and the color of the bar do not match (i.e., are incongruent), individuals are instructed to withhold a response (no-go condition) (Caldas et al., 2012). On congruent stimuli, the color of word is the same of the color-bar (i.e., without color-word interference), while on incongruent stimuli, the color of word is different from the color-bar. However, the meaning of word matches the color-bar in both cases. The interference created by the incongruent condition generates conflict in three different ways: (i) between the “word” meaning and “color of the bar”; (ii) between the “color of the word” and the “word” meaning; (iii) between the “color of the word” and the “color of the bar.” These semantic conflicts trigger a response conflict and consequently, a delay in response time (RT) (Caldas et al., 2012). In this sense, the mechanism of inhibition and selective attention behind the SCWT involves the need of suppression and/or filtering irrelevant and distract stimuli related to the manipulated conflicts (Pessoa et al., 2003). Attention can be defined as the neural mechanism to keep focus on a relevant stimulus, often associated with the neural processes of inhibition and selective attention involving the prefrontal cortex (Schrobsdorff et al., 2012). Also, it has been proposed that allocation of attention is a result from attention-shifting and response inhibition (Sylvester et al., 2003). In general, to keep focus on relevant information (i.e., selective attention) it is necessary to ignore or avoid irrelevant distractor elements (i.e., inhibitory control). On SCWT, a substantial effort is usually required of selective attention and inhibition because the responses to stimuli depend on these cognitive mechanisms, whether in identification of response type (i.e., GO or NOGO), or in inhibition to irrelevant elements (i.e., color-word different to meaning of word). Aditionally, the concomitant measure of neuroelectric responses during a cognitive task helps to uncover the underlying mechanisms of cognitive processes and performance and further helps to improve our understanding of this complex process (Luck, 2005).

Electroencephalography (EEG) is a non-invasive technique with high temporal resolution which can be used to assess neuroelectric responses time-locked to a stimulus (e.g., visual, cognitive, and tactile) or response (i.e., ERP, event-related potentials) and frequency bands related to a given state (e.g., resting or cognitive task) (Luck, 2005; Cheron et al., 2016). Finally, although the spatial resolution of EEG is low, inverse problem solutions can be used to estimate the cortical location where such cognitive processes might have happened (Denis et al., 2017). ERPs can be analyzed by their amplitude (i.e., peaking voltage in a time window), latency (i.e., time between the onset of the stimulus and the peak of a waveform) or area (i.e., mean activity in a time window) (Luck, 2005). When the task is GO/NOGO type of response, GO-incongruent condition responses present increased interference, and a negative wave around 200 ms (N200), related to conflict detection, is expected. N200 is normally evoked on frontocentral/anterior brain area, for instance on the anterior cingulate cortex (ACC) (Patel and Azzam, 2005). Decreased N200 amplitude and/or longer latency represents reduced ability to inhibit an inappropriate response and delayed processing speed (Hillman et al., 2009). On the other hand, a positive wave peaking around 300 ms (P300) can be identified in tasks that require attention and discrimination between different stimuli. P300 may also underlie attentional resources, like allocation, and is considered a measure of stimulus identification and classification speed (Pontifex et al., 2009). Due to the strong link with attention, P300 is frequently evoked by prefrontal cortex (Patel and Azzam, 2005) in which its amplitude has been associated with the quantity of attentional resources that are involved on the task, and a more pronounced response would be expected in incongruent stimuli (Patel and Azzam, 2005). In GO/NOGO tasks, the P300 latency is related to the timing in which attentional resources are recruited: incongruent trials (higher conflict) may have a delayed response compared to those for congruent trials (Ilan and Polich, 1999; Patel and Azzam, 2005). Despite the N200 and P300 being important components of neuroeletrical responses to SCWT, a more negative activity around 450 ms (N450) is the most prominent marker of Stroop conflict associated with conflict interpretation; this is more pronounced at frontocentral to centroparietal regions, especially by the ACC (Tillman and Wiens, 2011; Caldas et al., 2012; Ergen et al., 2014). Considering the enhanced magnitude of conflict detection present on incongruent stimuli, it is expected that the amplitude of N450 will be higher than that on congruent stimuli since the demand of neural resources for precise interpretation is also higher.

### Impact of Exercise in the Brain

A consistent body of literature has shown positive associations/effects of physical activities and exercise with brain function and structure (Colcombe et al., 2006; Chaddock et al., 2010a; Erickson et al., 2011; Hillman et al., 2011; Chang et al., 2012; Hyodo et al., 2012; Nagamatsu et al., 2012; Voss et al., 2013; Donnelly et al., 2016; Fontes et al., 2016), and that physical activity participation through the lifespan is beneficial to cognition (Hillman et al., 2008). These include a single bout of exercise increasing cognitive performance and ERP amplitudes in children (Hillman et al., 2011; Donnelly et al., 2016), adults (Chang et al., 2012), and elderly (Hyodo et al., 2012). Also, greater aerobic fitness (i.e., maximum oxygen uptake; VO_2__max_) was associated with greater hippocampal volume and long-term memory in children (Chaddock et al., 2010a), and exercise training with both aerobic and resistance exercise has shown to result in increased brain integrity and gray and white matter volume (Colcombe et al., 2006; Erickson et al., 2011; Nagamatsu et al., 2012; Voss et al., 2013; Fontes et al., 2016). Potential underlying mechanisms for exercise-induced improvement in cognitive function and brain structure include brain-derived neurotrophic factor (BDNF), involved in neuronal survival, differentiation, and synapse strengthening and formation; vascular endothelium growth factor (VEGF), involved in blood vessels growth and increased vascularity; insulin-like growth factor 1 (IGF-1) which has similar downstream signaling mechanisms to BDNF in synaptic plasticity; among others (Vaynman and Gomez-Pinilla, 2006; Voss et al., 2014). Studies with both acute (i.e., single session) and chronic (i.e., training program lasting several weeks/months) exercise have shown that increases in cognitive performance and/or brain structure are associated with these variables (Erickson et al., 2011; Tsai et al., 2016; Pensel et al., 2018). Interestingly, improvements in physical fitness measures are also positively correlated with enhancement in cognitive and structural adaptations (Erickson et al., 2011; Pensel et al., 2018). In addition, among different cognitive domains, the largest exercise-induced effects for cognition are executive-control processes (Colcombe and Kramer, 2003).

All the studies above were performed with sedentary or active individuals. Fewer studies have assessed cognitive and structural adaptations to sports training in athletes. These individuals take sports practice to a completely different level; this includes exercise intensity and volume as well as motor skills. Thus, if a linear dose-response relationship was drawn between exercise intensity/volume and cognitive neural adaptations, one could speculate that athletes could also benefit from greater improvements not only on physical but also cognitive/neural performance (Yarrow et al., 2009; Park et al., 2015; Pensel et al., 2018). Haier et al. (1988) proposed the “neural efficiency” theory that suggests individuals with higher cognitive ability presented lower energetic demand, after finding negative associations between brain metabolic rate and intelligence scores. This theory has been used to infer improved cognitive performance by the athletes. In sports, the neural efficiency hypothesis could be interpreted as individuals with higher sports expertise (i.e., advanced athletes) would present improved cognitive performance associated with similar or decreased neural activity, as already shown in karate and basketball players (Del Percio et al., 2008, 2009; Babiloni et al., 2010; Callan and Naito, 2014). However, studies have shown that athletes’ neuroelectric responses may change as a result of the cognitive task used (Cheron et al., 2016), such as greater amplitudes in P100 and P200 (i.e., early components related to stimulus detection and stimulus evaluation) in an oddball paradigm in skilled martial art athletes compared to novice athletes (Sanchez-Lopez et al., 2016) and greater alpha de-synchronization during balance control in expert karate athletes (Del Percio et al., 2007). Similarly, a recent review has shown that chronic exposure to exercise, such as during sports practice, increases amplitude and decreases latency on P300 and N200 (Cheron et al., 2016). In fact, the later affirmative can be conflicting with the neural efficiency since higher and faster recruitment of resources has been associated with sports practice. Therefore, applying both temporal and frequency EEG analysis in the present study may help to understand this conflict.

Additionally, athletes are more resilient to mental fatigue, a psychobiological state caused by prolonged exposition to a cognitively demanding task that has been shown to decrease physical performance (Van Cutsem et al., 2017). Martin et al. (2016) showed that professional cyclists had greater accuracy and a faster RT (i.e., greater inhibitory control and/or selective attention) compared to recreational cyclists in a selective attention task using only incongruent stimuli of the SCWT during 30 min used to induce mental fatigue. Importantly, mental fatigue did not affect professional cyclists’ performance on a 20 min cycling time trial, while it decreased the performance of recreational cyclists (Martin et al., 2016). Hence, one could speculate that athletes with longer experience may have not only a stronger body, but also a “stronger brain” (e.g., improved and/or differential activation to cognitive stimuli) (Jacini et al., 2009; Nakata et al., 2010; Sanchez-Lopez et al., 2014). Notwithstanding, it has been proposed that cognitive performance plays a crucial role in exercise performance and fatigue (i.e., exercise termination); however, this is ultimately up to the individual when they decide whether to continue struggling/fighting (i.e., the decision on to increase, decrease or maintain pace) or to stop exercising (Pageaux, 2014; Robertson and Marino, 2016; McMorris et al., 2018). Thus, cognitive adaptations might also be a result of sports training (i.e., either increased cognitive performance, lower neuronal demand, or greater resistance to mental fatigue).

In spite of the studies above, brain oscillations and their relationship with sports performance are relatively poorly understood, although it has been proposed that they play an important role in performance (Babiloni et al., 2010; Cheron et al., 2016; Denis et al., 2017; di Fronso et al., 2017; Wang et al., 2017). Although sport expertise may have an integrative perspective in the brain, including sensorimotor and cognitive control (Yarrow et al., 2009; Cheron et al., 2016), most studies were performed including an endurance sport (e.g., running/cycling) and sports with more technical-tactical dependence (e.g., badminton and tennis). Studies assessing contact sports (e.g., martial arts) that usually have a high demand to detect conflict information, decision making, and reaction time while simultaneously interacting with the opponent are relatively scarce, and this knowledge could help to understand the possible cognitive and electrophysiological characteristic that could differentiate novice from experienced athletes.

Judo, meaning the “gentle way,” is a centenary martial art created as a physical, mental, and moral pedagogy. Judo became a combat and Olympic sport whose purpose in the competitive context is to either throw or takedown an opponent to the ground, immobilizing or forcing the opponent to submit with a joint lock or a choke (Fortes et al., 2017). Judo requires great levels of muscle strength and power, for applying the throws, holding grips, or lock/choke, which depends not only on anaerobic but also aerobic fitness (Franchini, 2016). Expertise levels of judokas’ (i.e., judo athletes) are represented by the belt color, which ranges from white (WB; i.e., novice) to black belt (BB; i.e., experienced). These athletes also differ in physical performance, such as lower limb power and a peak torque of shoulder internal rotators (Detanico et al., 2016) and muscular endurance of the upper limbs (Vecchio et al., 2014). Additionally, judokas of different competitive levels (i.e., elite vs. non-elite) differ in judo-specific [e.g., a greater number of repetitions of judogi grip strength tests (Franchini et al., 2011a)] and non-specific [e.g., greater mean and peak power in a Wingate test (Franchini et al., 2005)] anaerobic power.

From a cognitive perspective, elite judokas (highly skilled senior and junior) presented better scores in the psychomotor skills (i.e., selective attention task and visual-motor coordination) than non-elite (less skilled senior and junior) (Supiński et al., 2014). The authors concluded that long-term judo training has positive effects on cognitive functions and also that high athletic performance in judo is conditioned to an optimal psychomotor ability (Supiński et al., 2014). Also, functional and structural differences have also been found in martial arts athletes (Jacini et al., 2009; Sanchez-Lopez et al., 2014). When compared to novices, skilled judo, taekwondo, and kung-fu athletes presented larger prefrontal positive activity and greater posterior negativity distribution before a motor response during a sustained attention task; novices showed a significantly larger response-related P300 in centro-parietal areas (Sanchez-Lopez et al., 2014). On the other hand, during the transient attention task, skilled athletes lacked prefrontal ability while the novice athletes showed strong prefrontal positive activity before a motor response and a large response-related P300 (Sanchez-Lopez et al., 2014). It was suggested that skilled athletes allocated simultaneously different processes in the sustained attention task due to the controlled attention and motor output required, while they used improved cue facilitation and controlled or more “automatic” responses in the transient task (Sanchez-Lopez et al., 2014). These results suggest that neuroelectric performance of novice and experienced/skilled athletes may depend on the task requirements. It is worth noting that the different neuroelectric responses occurred in the absence of a behavioral difference between groups for either accuracy or RT (Sanchez-Lopez et al., 2014). Interestingly, structural magnetic resonance imaging found greater gray matter volume in frontal, parietal, occipital, temporal lobes, and the cerebellum in 8 internationally competitive high-level professional judokas with at least 10 years of judo experience and averaging 5–6 h/day of training compared to healthy controls (Jacini et al., 2009).

Limitations of those previous studies comparing neuroelectric responses between experienced and novice athletes include the use of a channel-based analysis, which may result in a non-precise localization of the bioelectrical generator due to the conduction volume effect (Denis et al., 2017). Also, considering only ERPs can cause a loss of information about the very subtle changes in frequency bands, which may be noteworthy especially for sports performance (Babiloni et al., 2010; Cheron et al., 2016; Denis et al., 2017; Wang et al., 2017). In this regard, other approaches to assess neuroelectric responses to a cognitive stimulus may expand our understanding of how, when and where given cognitive processes occur. EEG frequency bands have been studied to understand how experienced individuals (i.e., professional of different areas) would differ from novices, with particular interest on theta (4–7 Hz), alpha (8–12 Hz), and beta (12–35 Hz) bands (Babiloni et al., 2010; Cheron et al., 2016; Denis et al., 2017; Wang et al., 2017). Previous experimental studies have shown that marksman exhibited overall higher theta and alpha, and lower beta and gamma (36–44 Hz) across various electrode positions before shooting and during processing of a verbal and spatial stimuli compared to novice shooters (Haufler et al., 2000). On the other hand, military personnel and police officers presented higher theta and lower alpha during judgment for using deadly force and decision making compared to civilians (Johnson et al., 2014). In another study, with great ecological validity, the alpha band predicted performance in professional soccer players of the Thailand’s team during the Women’s Asian Cup (Tharawadeepimuk and Wongsawat, 2017).

Moreover, event-related spectral perturbation (ERSP) is a time-frequency approach for EEG data analysis, time-locked to the event and highly frequency-band specific. ERSP involves the analysis of both event-related synchronization (ERS) and desynchronization (ERD) of EEG frequency bands during cognitive and visuomotor tasks (Babiloni et al., 2009, 2016; Del Percio et al., 2010; Wang et al., 2017). ERD has been suggested to reflect the activation and excitability of cortical neurons, while ERS has been linked to reduced information processing, low motor behavior and/or deactivation of cortical networks (Pfurtscheller, 2001). In addition, ERD could be interpreted differently according to the frequency band analyzed. For instance, mu rhythms are maximized during movement execution (Cebolla et al., 2015; Cheron et al., 2016) while reduction in amplitude of ERD in alpha band are linked to “arrest reaction,” that is, less ERD amplitude in more experienced athletes (Del Percio et al., 2011). Studies with ERSP have shown preliminary evidence that, for instance, expert golfers presented ERD for alpha and beta rhythms on the bilateral primary sensorimotor cortex before successful and unsuccessful putts (Babiloni et al., 2008). Furthermore, before striking the ball in successful putts, golfers presented suppression in high-alpha amplitude (10–12 Hz) over the frontal midline and the right primary sensorimotor area compared to unsuccessful putts (Babiloni et al., 2008). Also, in a case study with an Olympic air-pistol shooter, optimal performance for shooting was preceded by a lower ERD and higher ERS in theta in prefrontal and temporal areas, ERS in frontal areas in alpha and ERD in left temporoparietal areas in low alpha (8–10 Hz), and a clear ERS pattern in beta band in the right prefrontal and centroparietal areas (Di Fronso et al., 2016). Aside from precision sports, expert and non-expert tennis players present greater ERD in sensorimotor regions during action observation and anticipation in mu and beta bands, which are followed by greater accuracy on anticipation (Denis et al., 2017). Similarly, badminton expert players presented faster RTs and lowered RT variability (i.e., the standard deviation of RTs across trials) with similar ERS for theta and ERD for alpha and beta (Wang et al., 2017). On SCWT, differences on oscillatory behavior are mostly expected around 300–500 ms with higher desynchronization amplitude on alpha band. It is believed that this pattern is due to the higher attentional demands, semantic interpretation and elevated task difficulty usually evoked by frontal (i.e., ACC and dorsolateral prefrontal cortex) and parietal areas of the brain (Klimesch, 1999; Ergen et al., 2014). For athletes with long-term exposure to sports practice and competitions, it would be expected that neural dynamics are more efficient (i.e., less ERD amplitude) while creating a specific pattern of cognitive processing.

However, only a handful of studies have used the ERSP analysis regarding sport expertise in combat sports. Additionally, high-density of EEG channels associated with more precise of source localization methods (e.g., based on Independent Components Analysis, ICA) may provide not only a pattern of change, but also the place where this change may occur. Therefore, the present study aims to assess the possible differences in cognitive performance (i.e., accuracy and RT) as well as the neuroelectric responses (i.e., ERP, ERSP, and source location) and their respective neuroelectric generators during a selective attentional task between experienced (BB) and novice (WB) judokas. This information could help increase understanding of how practicing combat sports can modulate neural activities and may further help guide interventions that consider body-mind integration to enhance sports performance. Accordingly with previous literature, we have a three-way split hypothesis that, if the acquisition of expertise in judo is in part related to fundamental differences in the way these athletes selectively attend or inhibit irrelevant information on a context-free experimental cognitive task: (a) experienced judokas (BB) would present improved behavioral response (i.e., faster RT and/or greater accuracy) accompanied by comparable ERP and ERSP responses; (b) similar RT and/or accuracy between groups with BB judokas presenting larger N200, P300 and N450 wave amplitudes and/or shorter latency, and (c) BB judokas present less ERD amplitude for theta, alpha and beta bands, according to the “neural efficiency” hypothesis.

## Materials and Methods

### Study Design

In the first session, participants provided personal information regarding judo experience, training practices, and educational background (level and years), and underwent anthropometric measures. They also performed a cognitive test (i.e., Stroop Matching task for selective attention) with concomitant EEG measures. In the second session, participants performed a field test for estimating cardiorespiratory fitness (VO_2__max_) within a minimum 24-h interval. In the third session, participants performed a judo-specific fitness assessment (i.e., Special Judo Fitness Test; SJFT) within a minimum 48-h interval. Participants were asked to refrain moderate-to-vigorous physical activity in the 12 h preceding all testing sessions. All procedures and tools used in the present study are described in detail below.

### Participants

The study sample was composed by judokas divided into groups according to their experience level (i.e., belt color): 18 white belts (WB) judokas with up to 1 year of judo practice (age = 25.2 ± 5.8 years old, three women) and 16 black belts (BB) judokas with >10 years of judo experience (age = 26.5 ± 7.9 years old, two women). All participants were recreationally physically active (i.e., running and strength training), not under rapid weight loss strategies (e.g., dehydration) or competitive period.

Participants were recruited via folders in social media, judo training centers, and local competitions. Inclusion criteria were: (a) <1 year of judo experience (*for the WB group only*) or >10 years of judo experience (*for the BB group only*); (b) no previous experience in other martial arts or combat sports (e.g., Karate, Muay Thai, Jiu-jitsu, etc; *for the WB group only*); (c) present or past competitive participation in judo championships (*for the BB group only*); (d) not presenting with visual impairment that could affect performance on the cognitive task (e.g., colorblindness); (e) not presenting with injury that could impair physical performance in the tests. Exclusion criteria were: (a) data loss (e.g., poor data quality or equipment failure); (b) not attending to all study sessions; (c) lesion during the study and not able complete all experiments; (d) voluntary abandonment. Thirty-six individuals were screened. One did not meet the inclusion criteria (presented with colorblindness), and another suffered an injury during the physical test. Thus, the final sample was composed of 34 healthy judokas (five women) divided into groups as described above.

### Anthropometric Assessment

The anthropometric profile consisted of measuring the participant’s weight, height, body fat, and fat-free mass. Body mass (kg) and height were measured using standard procedures in the first session using an electronic scale with a stadiometer (Welmy^®^, W110H, Santa Bárbara d’Oeste, Brazil) with the participant wearing light clothes (appropriate for exercising). Body mass index was calculated as the ratio between body mass and height squared. Besides this, the dual-energy X-ray absorptiometry (DEXA; Lunar Prodigy, GE Medical System, Madison, WI, United States) scan was used to assess body composition (i.e., body fat and fat-free mass), again using standard procedures.

### General and Specific Physical Measures

We used two tests to estimate the athletes’ fitness. Cardiorespiratory fitness was estimated using the Yo-Yo Intermittent Recovery Level 1 Test (YY IR1), which consists of repeated 2 × 20-m runs back and forth between the starting, turning, and finishing points. The sprints are performed in a progressively increasing speed. The speed and distances covered at each speed was performed as follows: 10 km.h^–1^ (40 m), 12 km.h^–1^ (40 m), 13 km.h^–1^ (80 m), 13.5 km.h^–1^ (120 m), 14 km.h^–1^ (160 m), 14.5 km.h^–1^ (320 m), 15 km.h^–1^ (320 m), 15.5 km.h^–1^ (320 m), 16 km.h^–1^ (320 m), 16.5 km.h^–1^ (320 m), 17 km.h^–1^ (320 m), 17.5 km.h^–1^ (320 m), 18 km.h^–1^ (320 m), 18.5 km.h^–1^ (320 m), and 19 km.h^–1^ (320 m). After each sprint, there is a 10 s recovery to simulate intermittent sports activities. The running sprints and interval periods were controlled by audio beeps. The test was finished when the athlete was no longer able to follow the beeps two consecutive times. The total distance was determined as the distance covered (in meters) during the YY IR1, including the last 20 m run completed before failing to follow the beep sound two times consecutively. The test was performed on a sports court, and the 20-m distance was marked by cones. Maximum oxygen uptake (VO_2__max_) was estimated using the following equation: VO_2__max_ = [(total distance in meters × 0.0084) + 36.4]. The YY IR1 test was validated in athletes of intermittent sports against a direct measure of VO_2__max_ (*r* = 0.70) with excellent teste re-test reliability (*r* = 0.95; coefficient of variation = 8.7%) (Bangsbo et al., 2008).

**FIGURE 1:**
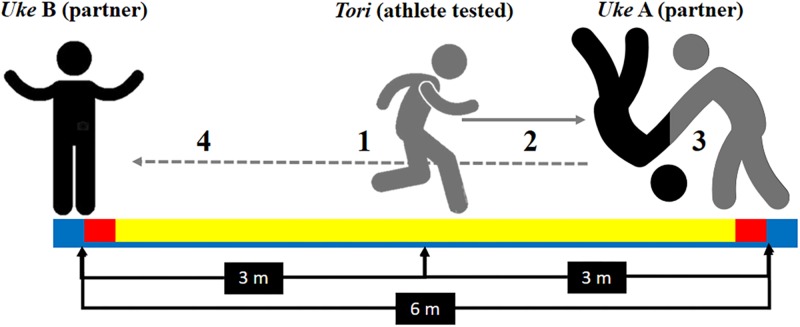
Special Judo Fitness Test representation (SJFT): (1) the tested athlete (*tori*) begins the test at the central point 3 m away from each throw partners (*uke*), who are 6 m apart from each other; (2) as soon as the experimenter shouted “go” the *tori* had to move toward one *uke* and (3) perform a throw using the *ippon-seoi-nage* technique; then (4) the *tori* had to move toward the other *uke* (6 m away) and, again, perform a throw using the *ippon-seoi-nage* technique. The purpose of SJFT is to perform as many throws as possible in three periods (15 s, 30 s, and 30 s) with 10 s interval.

We also used the Special Judo Fitness Test (SJFT) for the specific physical demands of judo (Sterkowicz, 1995). The athlete being tested (*tori*) begins the SJFT at the central point 3 m away from each throw partners (*uke*), who are 6 m apart from each other. As soon as the experimenter shouted “go” the *tori* had to move toward one *uke* and perform a throw using the *ippon-seoi-nage* technique. Then the *tori* had to move toward the other *uke* (6 m away) and, again, perform a throw using the *ippon-seoi-nage* technique (see Figure 1 for an SJFT layout). The purpose of SJFT is to perform as many throws as possible in three periods (15 s, 30 s, and 30 s) with 10 s interval. SJFT analyzes the anaerobic fitness represented as the number of a judo-specific throw (*Ippon Seoi Nage*) and aerobic fitness represented by the cardiovascular demand required to the judoka (i.e., heart rate at the end and 1-min following the SJFT) (Sterkowicz, 1995; Franchini et al., 1999, 2009), but the main contribution to SJFT has been shown to be from the alactic energy system (Franchini et al., 2011b). The overall performance in SJFT is calculated as an index; this includes the heart rate measured immediately at the end of SJFT (HR_final_) and 1 min after (HR at 1-min after) as well as the test number of throws performed during the entire test (NT) (Sterkowicz, 1995; Franchini et al., 1999, 2009). The SJFT index was calculated as follows: SJFT_index_ = [(HR_final_ + HR at 1-min after)/NT] (Sterkowicz, 1995; Franchini et al., 1999, 2009). HR was recorded continuously during the SJFT using an HR monitor (RS800cx, Polar Electro OR, Finland) with a 1000 Hz sampling rate. The HR data were downloaded by Polar Pro Trainer 5 (Polar, Finland) for further analyzes.

Both YY IR1 and SJFT were used because VO_2__max_ influences cognitive performance, brain structure and function (Colcombe et al., 2006; Hillman et al., 2008; Chaddock et al., 2010b; Erickson et al., 2011), which could be a confounding variable in the present study. Even though SJFT is associated with cardiorespiratory fitness, it does not allow estimating the VO_2__max_. Thus, we performed the YY IR1 for estimating VO_2__max_, which was used as a control variable. The YY IR1 and SJFT tests were performed on different days to avoid affecting the performance in each of them.

### Stroop Matching Task

**FIGURE 2:**
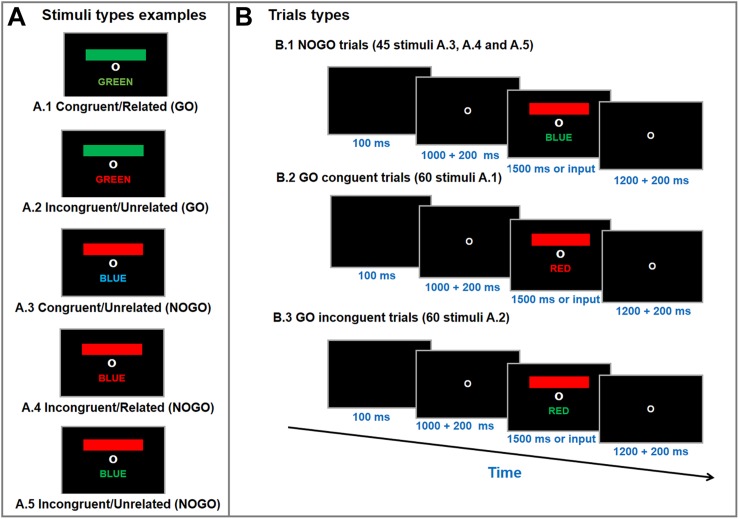
Stimulus type examples **(A)** and trial types **(B)** of the Go/NoGo Stroop Color-Word Matching test. Participants were asked to match color-bar with the color-name (i.e., GO trials; A1 and A2), irrespective of font color, as accurate and quick as possible. During the test a fixation circle appeared for 1200–1400 ms, followed by a blanc screen for 100 ms and another fixation circle for 1000–1200 ms. Then, a colored-bar and a color-name appeared on the screen for 1500 ms or until a response was entered (i.e., button press). The test was performed in three randomized blocks with 55 stimuli each (20 congruent, 20 incongruent, and 15 neutral per block).

A computerized version of the GO/NOGO Stroop Matching task was used to measure selective attention performance; a similar test setup has been described in detail in the literature (Caldas et al., 2012). Figure 2 displays the Stroop Matching stimuli types and test layout. The stimuli were a colored bar and a colored word. The bar, word and font stimuli could appear in red, green or blue (words were in Brazilian Portuguese). A black screen appeared for 100 ms, followed by a fixation screen for anywhere between 1000–1200 ms (random). Finally, the stimulus appeared on the screen until the participant entered a response or for 1500 ms. The inter-trial interval was a fixation screen 1200–1400 ms (random). The purpose of the test was to match the bar color with the name color (GO), irrespective of font color (Caldas et al., 2012). Participants were instructed to press the key “1” on a keyboard as soon as the stimuli appeared if the stimulus was GO type (i.e., bar color matched name color), and not to press any button if the stimulus was a NOGO type (i.e., bar color unmatched name color) (Caldas et al., 2012). Participants were told that the purpose of the task was to be as accurate and quick as possible. Stimuli and response markers were sent to the EEG amplifier via a parallel port by the stimulus presentation software (E-prime 2.0, Psychology Software Tools, Inc, Sharpsburg, PA, United States).

The combinations of colored bars and words allows for five different stimulus types: (a) congruent/related (i.e., colored bar, color-word meaning and color-font matched = GO); (b) incongruent/unrelated (i.e. colored bar, color-word meaning matched, but color-font did not = GO); (c) congruent/unrelated (i.e., color-word meaning and color-font matched but colored bar did not = NOGO); (d) incongruent/related (i.e., colored bar and color-font matched, but color-word meaning did not = NOGO); (e) incongruent/unrelated (i.e., unmatched colored bar, color-word meaning and color-font = NOGO) (Caldas et al., 2012). The stroop matching task used consisted of 60 congruent (GO), 60 incongruent (GO) and 45 neutral (NOGO) stimuli trials, which were divided into three blocks (20 congruent, 20 incongruent, 15 neutral per block) which were presented in a randomized order. The signal-to-noise ratio (SNR) for ERP increases linearly as the trial count increases logarithmically (Thigpen et al., 2017) so that an SNR recommended of ≥10 (Luck, 2005) is reached with 40 trials (Thigpen et al., 2017). Thus, using 60 trials for each main condition indicates high-quality data.

The Stroop test was performed on the E-prime 2.0 (Psychology Software Tools, Inc, Sharpsburg, PA, United States). The software recorded response input, from which accuracy was calculated (% of correct responses) and RT for correct responses in milliseconds for GO stimuli (behavioral performance). For the analysis, only GO stimulus correctly attended to, with RT > 200 ms (i.e., excluding anticipatory responses) and <1500 responses ms (i.e., excluding slow responses) were included. RT and accuracy were analyzed separately into the congruent and incongruent stimuli and the stimuli types (RT and accuracy) was compared within both groups. Also, RT variability (RTV) was calculated as the standard deviation of the RT for each stimulus type. Finally, the Stroop effect was calculated as the difference in RT between incongruent and congruent stimuli.

### EEG Recording and Pre-processing

Electroencephalography was recorded using 64 Ag-AgCl active electrodes (ActiCAP, Brain Products, Germany) covering the temporal, frontal, parietal, and occipital areas of the scalp, according to the 10–20 international system. The FCz and AFz electrodes were used as the reference and ground electrodes, respectively. Impedances were kept below 20 kΩ, and the sampling rate was 1000 Hz. The EEG signal was amplified by a BrainAmp MR (Brain Vision, Brain Products, Germany) and recorded with the Brain Vision Recorder (Brain Products, Germany). Stimuli and response markers were inserted into the continuous EEG data by the stimulus presentation software (E-prime 2.0, Psychology Software Tools, Inc, Sharpsburg, PA, United States) using a parallel port.

Offline processing was performed on the EEGLAB toolbox v.14.0.0 program (Delorme and Makeig, 2004) as follows: (a) low-pass filter of 55 Hz and high-pass filter of 1 Hz; (b) noisy channels were identified by kurtosis criteria and removed; (c) average reference; (d) segmentation according to with stimulus markers (i.e., congruent and incongruent) from -1000 ms to 2000 ms, from pre to post-stimulus, respectively; (e) blink and muscle artifacts were identified and removed using an independent component analysis (ICA) (Jung et al., 2000); (f) brain dipole sources were localized using the Equivalent dipole source localization of independent components algorithm (DIPFIT2) (Oostenveld et al., 2011).

Unpaired comparison between WB and BB groups were performed for congruent and incongruent stimuli using STUDY (Delorme and Makeig, 2004). Only dipoles located within the brain which had a maximum residual variance from the independent component scalp projection of 25% were kept. A feature vector comprising the 5–25 Hz power spectrum with equivalent dipole position and independent components scalp projection was built for each participant, followed by a dimensionality reduction via principal components analysis (PCA) to cluster independent components from multiple participants. The first 10 principal components were used as inputs to an artificial neural network clustering algorithm. Components located more than two standard deviations from cluster centers were labeled as outliers and therefore excluded. When a cluster had more than one independent component per participant, we chose the one presenting the smallest residual variance from the independent component scalp projection. Only clusters located at or above the cingulate cortex were considered the anatomical and methodological (i.e., dipole location) methods (Cohen et al., 2011) and given that the EEG is highly influenced by cortical brain tissue (Teplan, 2002). The cluster was only considered in the subsequent analyses if there were components of 50% + 1 of each group (nine components of BB and 10 components of WB). Additionally, Talairach coordinates (x, y, and z) of the averages of each cluster were used to identify the Brodmann areas (Lancaster et al., 2000). It is important to note that all other analysis steps were source-localization-oriented; in other words, ERP and ERSP were analyzed considering the source, not the channel, space. The EEG data analysis flow chart is presented in Supplementary Figure S1.

### ERP Data Processing

The preprocessed segmented EEG data was used for assessing the ERP for the congruent and incongruent trials considering 200 ms pre to 850 ms post-stimulus. Only stimuli followed by correct response markers were included in the analysis. Baseline correction was applied using −200 to 0 ms prestimulus period. The latency and amplitude for each ERP were assessed as follows: N200, maximum negative deflection between 150 and 300 ms; P300, largest positive-going peak within 250 to 500 ms poststimulus; N450, mean activity per spectral line from 350 to 500 ms poststimulus. This study used a data-driven analysis and, thus, ERPs were analyzed in the source-space, not channel-space (i.e., using the clusters sources). The ERP data has been shown to be highly consistent regarding peak voltage at individual time points and sensors [Cronbach’s alpha (α) >0.7 with 20 trials and >0.8 with 40 trials], voltage topography for each time point (α > 0.7 with 30 trials and >0.9 with ≥40 trials), voltage time course for each sensor (α > 0.7 with 40 trials and >0.9 with 80 trials), and also after averaging across time points and sensors (α > 0.7 with 20 trials and 8 electrodes and >0.9 with 20 trials and 15 electrodes) (Thigpen et al., 2017).

### Time-Frequency Analyses

We assessed the ERSP for time-frequency analyses, which is a measure of average dynamic changes in the amplitude of the broad EEG frequency band spectrum as a function of time relative to an experimental event. After preprocessing the continuous raw EEG data, as described above, the artifact-free individual segments of congruent and incongruent GO trials from −1000 ms pre-stimulus to 2000 ms post-stimulus were used for ERSP analysis. The 1000 ms preceding each stimulus type were defined as the baseline at stimulus base (e.g., fixation screen). The frequency spectrum was obtained with 3-cycle Morlet wavelets. A non-parametric permutation method with 2000 surrogate data sets was used for masking the ERSP plots for significance. The frequency bands were defined as theta (4 – 7 Hz), alpha (8 – 14 Hz), beta (15 – 30 Hz) (Ergen et al., 2014). ERSP was assessed in the source-space not channel-space (i.e., using the clusters sources). The results are presented a colored-contrast presenting changes post-stimulus in frequency band power relative to the baseline. Decreases and increases are represented in blue red, respectively. All analyses were conducted in EEGLAB.

### Data Analysis

The data distribution was assessed using the skewness and kurtosis. A two-tailed unpaired *t*-test was used for comparing sociodemographic (age and education), body composition (body mass, height, muscle mass, and percentage of body fat), aerobic fitness-related (predicted VO_2__max_, distance covered, and maximum, running speed), and SJFT parameters (number of throws, HR_final_, HR after 1 min, and STJF index) between groups. Cohen’s *d* was used as a measure of the effect size (ES) for pairwise comparisons. ES magnitude was qualitatively assessed as follows: trivial (<0.2), small (0.2 to 0.5), moderate (0.6 to 1.1), large (1.2 to 1.9), very large (2.0 to 3.9), and nearly perfect (≥4.0). Also, Pearson’s product moment correlation was used to assess the correlation between SJFT and aerobic fitness-related parameters as described above.

Further, Levene’s and Muchly’s tests were used to assess the homogeneity and sphericity of the data. Greenhouse-Geisser Epsilon correction was used when necessary. A 2 × 2 mixed ANOVA was used for comparing outcomes measures: accuracy, RT, RT variability, peak and latency for N200, P300, area for N450. The group (WB vs. BB) and stimuli type (congruent vs. incongruent) were included as factors for comparison. Fisher LSD’s *post hoc* test was used for identifying punctual differences. Partial eta squared (η^2^_p_) was used as a measure of ES for ANOVA. The magnitude of η^2^_p_ was classified as follows: small (0.01–0.059), medium (0.06 to 0.139), or large effect (≥0.14) (Cohen, 1973). Moreover, bootstrapped significance tests were used for testing single independent component ERSPs followed by the false discovery rate (FDR) for avoiding false-positives using the EEGLAB.

Finally, analysis of covariance (ANCOVA) was used to determine whether there are any significant differences between WB and BB on the dependent variable: ERPs (N200, P300, and N450) and ERSP (theta, alpha, beta), adjusted for the covariate (i.e., VO_2__max_). Statistical significance was set as *P* ≤ 0.05 for all analyses.

## Results

### General Characteristics and Physical Performance

Participants of both groups were relatively homogeneous with no significant difference in age, years of study, and body composition, though a moderate effect size was found for greater muscle mass for the BB group (Table 1). Concerning physical performance, the BB group covered a larger distance and reached a higher maximum running speed and predicted VO_2__max_ on the cardiorespiratory fitness test, all with a moderate ES. On the SJFT, the BB also presented a greater overall number of throws and lower SJFT index (i.e., better performance), with a large ES as compared to WB group (Table 1). No other significant difference was found. Moreover, considering that only five women were included (two in the BB and three in the WB group) we performed an ancillary analysis removing women, but the results were not different from the ones presented in Tables 1, 2 (see Supplementary Table S1).

**TABLE 1:** General characteristics of experienced and novice judo athletes regarding body composition, aerobic fitness, and judo-specific fitness.

	**Black belt (*n* = 16)**	**White belt (*n* = 18)**	***t***	***P***	**Cohen’s *d***	**Magnitude**
**Sociodemographics**						
Age (years)	26.5 ± 7.9	25.2 ± 5.6	0.571	0.671	0.18	Trivial
Education (years)	19.6 ± 4.2	18.6 ± 3.0	0.688	0.499	0.27	Small
**Body composition**						
Height (cm)	172.25 ± 8.12	171.39 ± 9.02	0.021	0.773	0.10	Trivial
Body mass (kg)	72.0 ± 13.7	68.61 ± 12.4	0.251	0.455	0.25	Small
Percent of body fat (%)	18.62 ± 6.14	22.28 ± 8.75	1.328	0.195	0.48	Small
Muscle mass (kg)	54.88 ± 9.63	49.42 ± 7.28	1.748	0.091	0.63	Moderate
**Aerobic fitness test (intermittent running)**						
Distance covered (m)	851.42 ± 462.08^∗^	533.75 ± 204.44	2.490	0.019	0.78	Moderate
Maximum running speed (km.h^–1^)	14.75 ± 0.82^∗^	14.18 ± 0.40	2.418	0.022	0.87	Moderate
Maximum oxygen uptake (ml.kg^–1^.min^–1^)	43.55 ± 3.81^∗^	40.71 ± 1.81	2.490	0.019	0.93	Moderate
**Special judo fitness test (SJFT)**						
Number of throws in the SJFT	25.33 ± 2.09^∗^	21.72 ± 2.49	4.004	0.001	1.57	Large
Final heart rate (bpm)	177.8 ± 10.43	180.9 ± 13.42	0.665	0.512	0.25	Small
Heart rate after 1 min (bpm)	155.26 ± 12.33	155.81 ± 20.74	0.085	0.933	0.03	Trivial
SJFT index (au)	13.29 ± 1.43^∗^	15.55 ± 1.96	3.079	0.003	1.31	Large

*^∗^Significant difference in favor of Black Belt group.*

**TABLE 2:** Comparison of RT and accuracy within the groups, accuracy, response time, and response time variability of the congruent and incongruent stimulus of the Stroop Test between experienced (black belt; *n* = 16) and novice (white belt; *n* = 18) judo athletes.

**Color belt**	**Variable**	**Congruent**	**Incongruent**	***F***	**df**	***t***	***P***	***d***	**Magnitude**
Black	Accuracy (%)	99.6 ± 0.9	93.3 ± 8.6	8.46	1,30	–	0.007	1.0	Moderate
	Response time (ms)	674.1 ± 24.2	826.2 ± 27.2	14.84	1,30	–	0.001	5.90	NP
White	Accuracy (%)	97.5 ± 7.3	97 ± 3	0.08	1,30		0.768	0.08	Trivial
	Response time (ms)	652.5 ± 22.8	801.9 ± 25.6	22.36	1,30	–	0.000	6.16	NP

**Stimulus type**	**Variable**	**Black belt**	**White belt**	***F***	**df**	***t***	***P***	***d***	**Magnitude**

Congruent	Accuracy (%)	99.6 ± 0.9	97.5 ± 7.3	0.11	1,32	–	0.742	0.40	Small
	Response time (ms)	674.1 ± 24.2	652.5 ± 22.8	0.26	1,32	–	0.873	0.91	Moderate
	Response time variability (ms)	119.7 ± 9.2	123.6 ± 8.7	0.51	1,32	–	0.994	0.43	Small
Incongruent	Accuracy (%)	93.3 ± 8.6	97 ± 3	0.11	1,32	–	0.742	0.44	Small
	Response time – incongruent (ms)	826.2 ± 27.2	801.9 ± 25.6	0.26	1,32	–	0.873	0.92	Moderate
	Response time variability (ms)	147.1 ± 7.1	150.8 ± 6.7	0.51	1,32	–	0.994	0.53	Small
	Stroop effect (ms)	152.1 ± 48.5	149.4 ± 50.3	–	–	0.15	0.878	0.05	Trivial

*±, standard deviation; *d*, Cohen’s *d*; NP, nearly perfect.*

Interestingly, no measure of SJFT (i.e., number of throws, final heart rate, heart rate after 1 min, or SJFT index) was correlated with measures of the maximal running test (i.e., distance covered, maximum running speed, and VO_2__max_ or predicted). The number of throws presented the highest correlation with SJFT index (*r* = −0.83; *P* < 0.001). All results from correlation analysis are displayed in Supplementary Table S2.

### Behavioral, Cognitive Performance

Table 2 shows the results of the comparison within and between groups for accuracy, RT and RT variability of the congruent and incongruent trials, as well as the Stroop interference. The stimulus types showed interaction on RT within both groups. On accuracy, an interaction was found only for the BB group. There was no main effect of group (all *P*s > 0.1) as well as group x stimuli type interaction (note F, df and P on Table 2 are for interaction only). Also, no significant difference was found for Stroop interference between groups.

### Source Localization (Component Clustering)

**FIGURE 3:**
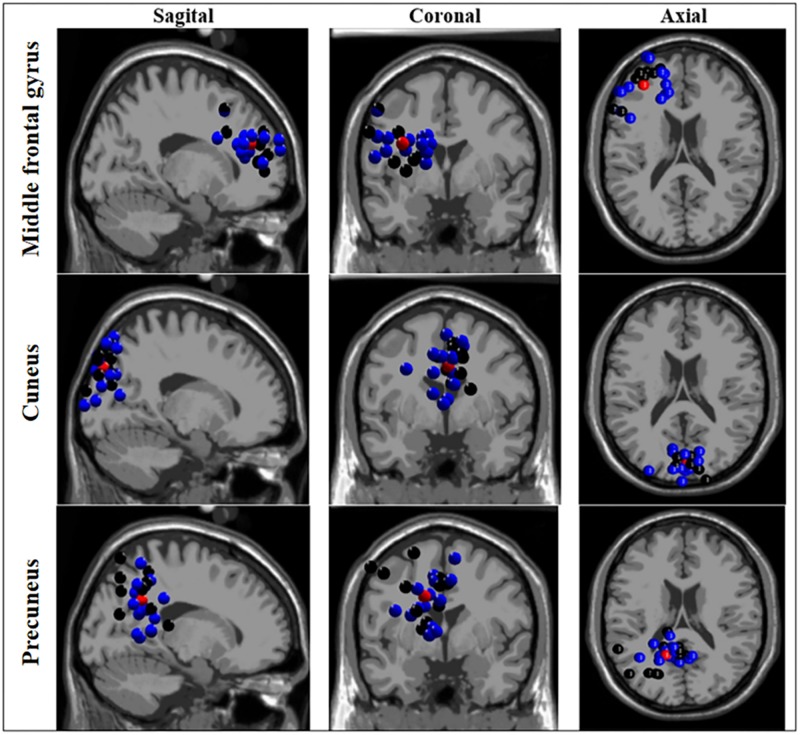
Source localization based on independent component analysis (ICA) of clusters for black and white belts (WB) judokas. Sagittal (left column), coronal (middle column) and axial (right column) views of clusters localized in the middle frontal gyrus, cuneus, and precuneus. Black and blue dots represent black and white belt judokas, respectively. The red dots represent the centroid location.

The ICA-based source localization applied to each group individually found clusters at the left anterior prefrontal cortex (Brodmann area 10), left dorsolateral prefrontal cortex (Brodmann area 9), orbitofrontal cortex (Brodmann area 11), left anterior cingulate cortex (Brodmann area 24), and right cuneus and left precuneus (both at Brodmann area 7) for the BB group. Relatively similar clusters were found the WB group such as the left anterior prefrontal cortex (Brodmann area 10), left cuneus (Brodmann area 19), bilateral precuneus (Brodmann area 39 and 31, for right and left, respectively), with the exception of bilateral premotor cortex (Brodmann area 4) which found clusters only for the BB group. As displayed in Figure 3, when the entire sample was analyzed as a whole, (both BB and WB groups) the ICA-based source localization found clusters at the left middle frontal gyrus (Brodmann area 10), left cuneus (Brodmann area 7), and left precuneus (Brodmann area 31). The detailed information regarding the clusters, centroid locations number of components, Brodmann area and Talairach coordinates for separate and grouped source localization analysis may be found in Supplementary Tables S3, S4.

### ERP Responses

Table 3 displays the comparison of N200 and P300 peak and latencies as well as N450 area information for congruent and incongruent trials in each cluster between BB and WB groups. For congruent trials, the BB group showed greater P300 peak in the middle frontal gyrus compared to the WB, with the main effect of belt color with large ES (η^2^_p_ = 0.31). A significant stimulus type x belt color interaction with large ES (η^2^_p_ = 0.21) was found for incongruent trials, with the BB group presenting greater N200 peak in cuneus. On the other hand, a significant stimulus type x belt color interaction with large ES (η^2^_p_ = 0.17) for N450 area, but only a trend toward significance (*P* = 0.07) for lower N450 area incongruent compared to congruent trials in the WB group.

**TABLE 3:** Comparison of event-related potential for congruent and incongruent stimuli of Stroop Color-Word Matching Task between experienced (black belts; *n* = 16) and novice (white belts; *n* = 18) judo athletes.

			**Congruent**		**Incongruent**		***F***		
			**Black belts**	**White belts**	**Black belts**	**White belts**	**Belt**	**Stim**	**Int**
Middle frontal gyrus	*N200*	Peak (μV)	0.076 ± 0.15	0.023 ± 0.04	−0.016 ± 0.13^#^	−0.022 ± 0.06^[*dollar*]^	0.61	8.64^∗^	0.97
		Latency (ms)	212.8 ± 62.04	218.4 ± 49.37	201.5 ± 59.37	220.6 ± 49.37	0.35	0.61	0.97
	*P300*	Peak (μV)	0.180 ± 0.23	0.017 ± 0.04^[*dollar*]^	0.078 ± 0.15	−0.001 ± 0.03^[*dollar*]^	9.00^∗^	2.46	1.13
		Latency (ms)	422.3 ± 90.66	362 ± 89.84	396.7 ± 97.03	389.2 ± 98.81	1.07	0.00	1.13
	*N450*	Area (μV)	0.24 ± 0.35	0.067 ± 0.08	0.071 ± 0.33^#^	0.03 ± 0.05^[*dollar*]^	1.36	9.74^∗^	4.00
Cuneus	*N200*	Peak (μV)	0.028 ± 0.09	0.005 ± 0.07	0.049 ± 0.11	−0.022 ± 0.08^#&^	0.11	1.79	5.97^∗^
		Latency (ms)	227.5 ± 58.7	225.2 ± 56.6	228.4 ± 57	235.2 ± 51	0.01	0.34	0.24
	*P300*	Peak (μV)	0.001 ± 0.02	0.000 ± 0.02	0.006 ± 0.03	−0.011 ± 0.03	0.75	0.40	2.47
		Latency (ms)	322.3 ± 90.1	338 ± 77.1	328.3 ± 62.6	352.4 ± 85.7	0.39	1.08	0.17
	*N450*	Area (μV)	0.081 ± 0.13	0.097 ± 0.18	0.107 ± 0.14	0.059 ± 0.18^#^	0.06	0.16	4.38^∗^
Precuneus	*N200*	Peak (μV)	−0.071 ± 0.13	−0.079 ± 0.06	−0.041 ± 0.14^#^	−0.018 ± 0.06^#^	0.03	20.1^∗^	2.31
		Latency (ms)	207.6 ± 50.8	187.6 ± 30.4	204.9 ± 42.7	188.4 ± 33.2	1.37	0.14	0.45
	*P300*	Peak (μV)	−0.016 ± 0.04	−0.02 ± 0.05	−0.021 ± 0.06^#^	−0.027 ± 0.03^#^	0.01	1.30	0.14
		Latency (ms)	401.3 ± 86.9	335.5 ± 72.9	384.3 ± 72.6	316.7 ± 62.5^[*dollar*]^	4.18^∗^	3.25	0.02
	*N450*	Area (μV)	0.147 ± 0.17	0.162 ± 0.24	0.16 ± 0.15	0.114 ± 0.21	0.04	0.43	1.27

*^∗^, significant effect (all *P*s < 0.05); ^#^, different from congruent stimulus within the same group (all *P*s ≤ 0.05); ^$^, different from congruent stimulus compared to the black belt group (all *P*s ≤ 0.05); ^&^, different from incongruent stimulus compared to the black belt group (all *P*s ≤ 0.05).*

**FIGURE 4:**
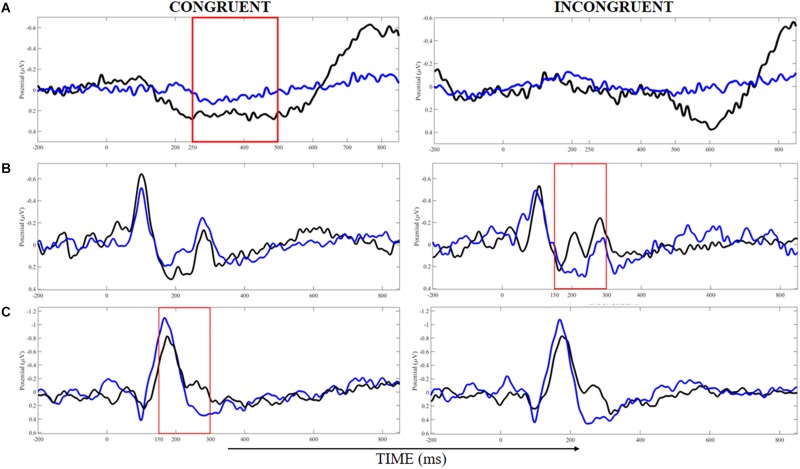
Analysis of event-related potentials in clusters localized at the middle frontal gyrus **(A)**, cuneus **(B)**, and precuneus **(C)** for congruent and incongruent trials in white (blue lines) and black (black lines) belts judokas. Areas demarked with red indicated the time-windows where the significant difference was found (*p* < 0.05).

Finally, we found a significant main effect of belt color for P300 latency on the Precuneus, with large ES (η^2^_p_ = 0.15), and the trend toward significance for the main effect of stimulus type (*P* = 0.09) with moderate ES (η^2^_p_ = 0.12). *Post hoc* test showed a significantly shorter P300 latency for incongruent compared to congruent trials in the WB group, and also a trend toward significance (*P* = 0.060) for a longer P300 latency on the Precuneus for incongruent trials of the BB group compared to the incongruent trials of the WB. Other main effects are reported in Table 3. Figure 4 showed the time-windows where the differences are present.

### ERSP

**FIGURE 5:**
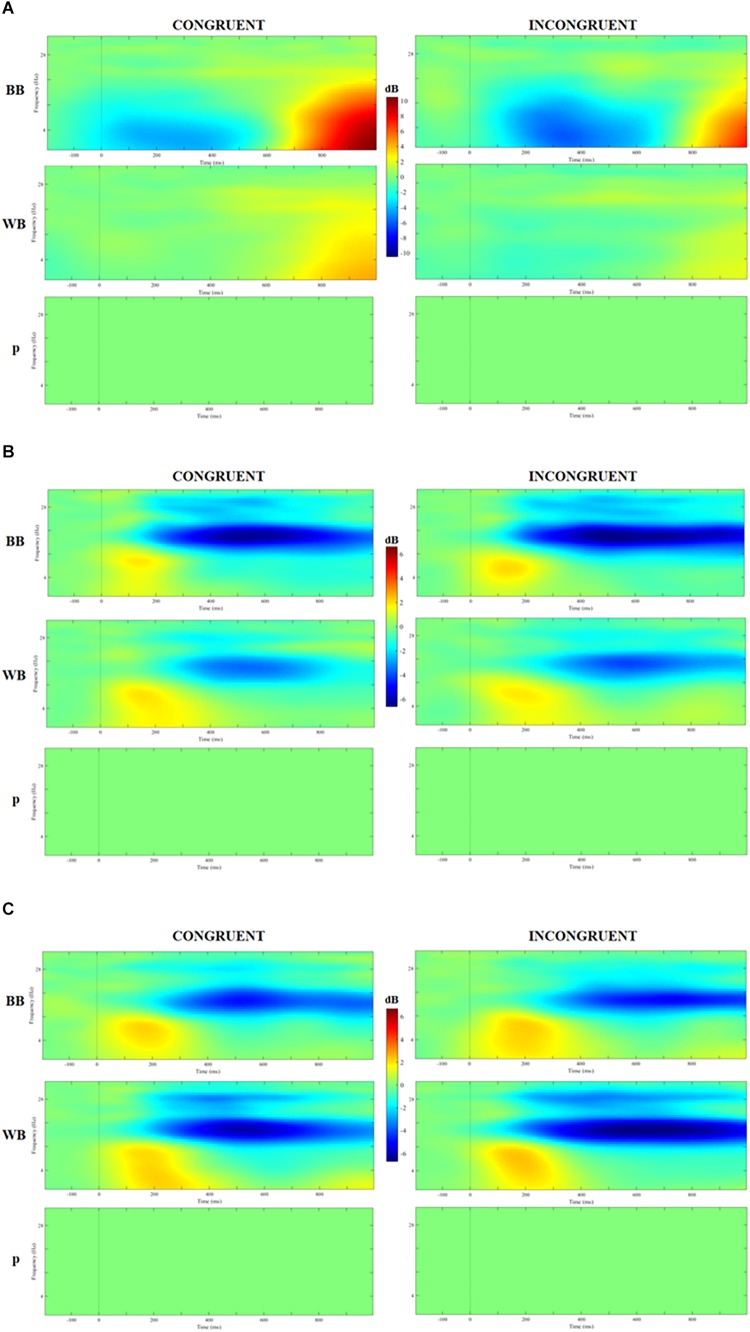
Analysis of event-related spectrum potential (ERSP) in clusters localized at the middle frontal gyrus **(A)**, cuneus **(B)**, and precuneus **(C)** for congruent and incongruent trials in WB and black belts (BB) judokas and the results of 2 × 2 ANOVA with false discovery rate (FDR) correction.

Figure 5 displays the time-frequency analysis comparison between groups. No significant difference nor interactons was found for theta, alpha or beta band for congruent or incongruent trials (all *P*s > 0.05).

### Covariance Analysis

ERP data in which significant differences were found for peak, latency and/or area between the groups were compared using an analysis of covariance using VO_2__max_ as a covariable, given its influence on brain structure and function (Colcombe et al., 2006; Hillman et al., 2008; Chaddock et al., 2010b; Erickson et al., 2011). None of the ERPs were significantly influenced by VO_2__max_ at the middle frontal gyrus [*congruent trials*: N200 peak: *F*(1,19); *p* = 0.397, P300 peak: *F*(1,19); *p* = 0.840, N450: *F*(1,19); *p* = 0.225; *incongruent trials*: N200 peak: *F*(1,19); *p* = 0.620, P300 peak: *F*(1,19); *p* = 0.185, N450: *F*(1,19); *p* = 0.168]; cuneus [*congruent trials*: N200 peak: *F*(1,21); *p* = 0.914, N450: *F*(1,21); *p* = 0.762; *incongruent trials*: N200 peak: *F*(1,21); *p* = 0.894, N450: *F*(1,21); *p* = 0.476]; precuneus [*congruent trials*: N200 peak: *F*(1,22); *p* = 0.286, P300 peak: *F*(1,22); *p* = 0.399, P300 latency: *F*(1,22); *p* = 0.658; *incongruent trials*: N200 peak: *F*(1,22); *p* = 0.301, P300 peak: *F*(1,22); *p* = 0.724, P300 latency: *F*(1,22); *p* = 0.301].

## Discussion

We verified whether cognitive performance and electrophysiological responses to a selective attentional task would be influenced by expertise level in judo. No difference for behavioral performance (i.e., RT, RTV, and accuracy) was found between BB and WB judokas. Component clustering from the Stroop Task resulted in three centroids located at middle frontal gyrus, cuneus, precuneus, from which ERP and ERSP responses were analyzed. No significant difference was found in ERSP for theta, alpha and beta band between groups. Finally, ERP responses showed some contradictory results, which do not fully confirm nor reject our hypothesis. Namely, the BB group displayed greater P300 in the middle frontal gyrus, which is indicative of greater attentional resource allocation capacity. Also, the BB group showed a greater N200 peak in cuneus, which is an indicator of greater conflict detection. Finally, we found a trend toward significance (*P* = 0.07) for lower N450 area (more negativity) in cuneus for incongruent compared to congruent trials in the WB group, which may indicate the expected higher demand for neural resources in such trials, despite no difference in the BB group. However, P300 latency was shorter in the incongruent trials of the WB group compared to both congruent (significant) and incongruent trials of the BB group (trend toward significance, *P* = 0.06), also the congruent trials in the WB group (trend toward significance, *P* = 0.07) in the precuneus. In other words, the WB group displayed the fastest attentional resource allocation for incongruent trials. Therefore, our findings rejected hypothesis “a” and “c” since no behavioral advantage was found for the BB, and only partially supports hypothesis “b,” which was that similar RT and/or accuracy between groups with BB judokas presenting larger N200 and P300 peaks and/or shorter latency (Wang et al., 2017), and greater ERS for theta band, ERD for alpha band and beta bands (Denis et al., 2017; Wang et al., 2017). However, only ERP and part of them were confirmatory of advantage in favor of experienced judokas. These findings expand on the literature showing that higher expertise in judo does not necessarily imply fundamental differences in the way these athletes selectively attend to or inhibit irrelevant information on a selective attention task involving semantic conflict, when compared to novice judokas.

Our results opposed to the finding of previous studies showing that individuals with greater physical fitness present lower N200 amplitude (Themanson and Hillman, 2006; Stroth et al., 2009). Despite this, a recent review has shown that increase in N200 amplitude has been associated with the effect of chronic exercise exposure. That is, long-term exercise could modulate the capacity of conflict detection (Cheron et al., 2016). Additionally, the P300 is involved in the allocation of attentional resources and stimulus discrimination (Patel and Azzam, 2005; Taliep et al., 2008). Many studies have indicated that the components of attention (included P300) present larger amplitude in athletes than non-athletes (Jin et al., 2011; Cheron et al., 2016; Sanchez-Lopez et al., 2016), which was also found in our results. However, the BB group showed slower latency in P300 than WB in discordance to studies that showed the opposite (Taliep et al., 2008; Sanchez-Lopez et al., 2016). The novel finding of the present study is that experienced judokas also present lower conflict processing, as the BB group showed lower N450 area (more negative) for incongruent trials, while the WB group showed no difference. The conflict generated by the SCWMT results in greater negativity around 450 ms after stimulus onset, even for congruent stimulus (Caldas et al., 2012). Taken together, the variation of the neural electrophysiological activity among the studies may represent a specific adaptation imposed by each sports training demand, especially in martial arts in which attentional resource allocation can interfere with the successful decision-making process during combat (Sanchez-Lopez et al., 2014, 2016).

A possible explanation for the present results can be the cognitive task used, which certainly influenced both behavioral performance and ERP and ERSP neuroelectric responses. Denis et al. (2017) used an anticipation task with video stimulus that had a tennis coach moving and hitting the tennis ball toward the participants, that had to respond the shot direction. They found greater behavioral performance (anticipation accuracy) and a greater ERD on the mu frequency (11–13 Hz) on the bilateral sensorimotor cortex (Denis et al., 2017). In the present study, we did not find behavioral nor ERSP differences between the expertise groups. In the Denis et al. (2017) study, they used a sport-specific stimulus, which required greater levels of expertise from individuals to succeed. In this case, experts found both greater behavioral and improved neuroelectric responses as compared to a novice, considering the movement anticipation required from the task. In the present study, we used a non-specific cognitive task which may have hindered finding of a possible difference between groups.

On the other hand, one may argue that other studies such as Wang et al. (2017) showed better behavioral performance (RT but not accuracy) in badminton players compared to athletic controls using a Flanker arrow test. However, neither ERP nor ERSP was different between groups (Wang et al., 2017). The conflicting behavioral performance may be due to the case that although Wang et al. (2017) did not use a sport-specific, but a general selective attentional test (Flanker task), it differs considerably from the task used in the present study. In the Flanker, task participants are required to determine the direction of the center arrow, which requires ignoring flank arrows, especially in the incongruent trials. In the present study, we used a Stroop Matching Go/NoGo task. We believe the different results may be explained by the fact that the Flanker task is more closely related to badminton practice than SCWMT is to judo. Also, the Flanker task involves only selective attention and inhibitory control, while the SCWT involves semantic conflict which requires greater cognitive processing as compared to the Flanker task. In fact, the RT and RTV of Wang et al. (2017) was about half of the average values found in the present study.

It is interesting to note that the behavioral results of the present study align with previous studies (Sanchez-Lopez et al., 2014, 2016). Similar to the present study, Sanchez-Lopez et al. (2016) did not find a difference in accuracy or RT in sustained or transient attention tasks between skilled and novice judo, taekwondo, and kung-fu athletes. However, they found that only skilled athletes presented P100 and P200 differences between the target and non-target conditions, while only novice athletes showed differences in N200 (Sanchez-Lopez et al., 2016). The difference between groups occurred for frontal (mainly in the superior frontal gyrus and medial frontal gyrus) and limbic lobes (mainly in the ACC) (Sanchez-Lopez et al., 2016). Similarly, we also found component clusters at the middle frontal gyrus, where the greatest P300 peak was found for congruent trials in the BB. This confirmed a greater allocation of attentional resources, stimulus identification and processing speed in the experienced group (Pontifex et al., 2009). Likewise, Sanchez-Lopez et al. (2014) also did not find a difference in accuracy or RT in an oddball paradigm between skilled and novice judo, taekwondo and kung-fu atheltes, but with different patterns of neuroelectric activity. The Sanchez-Lopez et al. (2014) study assessed martial arts athletes using a relatively similar task as the one used by Wang et al. (2017), with a sample of badminton athletes, and the absence of behavioral difference reinforces our hypothesis that a difference in behavioral outcomes between experienced and novice individuals may appear only with sport-specific cognitive task and/or a task that closely relates to the sport in question. It is important to note that the comparative “athletic control” group in Wang et al. (2017) study was also composed by athletes from track-and-field and dragon boat athletes with a professional training experience of at least 7 years. Therefore, it is likely that better performance in individuals with higher sports expertise may be limited to sports-specific stimulus and not differences in the way these athletes selectively attend or inhibit irrelevant information on a context-free experimental cognitive task.

With the advances in neural processing methods, the source detection through ICA has been suggested to provide a more precise activity localization than channels (Makeig et al., 1995; Delorme et al., 2012). In the present study using ICA, both groups presented increased activity in middle frontal gyrus, cuneus, precuneus during the cognitive task (see Supplementary Tables S3, S4), which was expected, considering the involvement of frontal and parietal cortices on cognitive tasks (Hillman et al., 2008; Li et al., 2009). However, the expert group presented clusters in the cingulate gyrus and middle, superior, medial frontal gyrus, while the non-expert group presented clusters in the precentral gyrus in both sides. One possible way to interpret this data is that individuals with longer training experience adapt to use fewer attentional resources to perform a given task with an improved inhibitory capacity of irrelevant cues (Milton et al., 2007; Yang, 2015). In fact, conflict resolution is intrinsically related to the decision-making process in martial arts (Sanchez-Lopez et al., 2016) and long-term practice may benefit not only brain morphology (Jacini et al., 2009), but also brain function (Callan and Naito, 2014; Kim et al., 2014). Nevertheless, only the non-expert group (i.e., WB) presented increased activation in the precentral cortexes, which is involved in motor planning, production and movement-related decision-making (Ojakangas et al., 2006), which may indicate that expert athletes have a functional neural adaptation to perform a cognitive-motor task.

The ERSP is an interesting measure of the dynamic changes in the amplitude of the large EEG frequency spectrum about the stimulus since it allows an analysis of both frequency and time domains (Makeig, 1993). However, after adjusting for spurious results, there was no significant change in ERSP in any frequency band between groups. These results are in line with Wang et al. (2017) who found no difference in ERSP between badminton players and athletic controls, despite better behavioral performance (RT but not accuracy) in using a Flanker arrow test groups (Wang et al., 2017). On the other hand, it opposes Denis et al. (2017) who found greater ERD on the mu frequency (11–13 Hz) on bilateral sensorimotor cortex associated with better greater behavioral performance in an anticipation accuracy tennis task, which could represent better ability for anticipating actions when compared to novice athletes (Denis et al., 2017). The reasons for the different results were explained above. In addition, there may have been a role of desynchronization in the theta band in coding and constructing the response to the stimulus (Cheron et al., 2016), the alpha band (cognitive control and reflects cortical inhibition control) (Cheron et al., 2016), and the beta band (movement execution and observation) (Babiloni et al., 2016). This seems to be particularly important for the decision-making process during a sports activity, considering the constant high load of technical and tactical information processing during both training and competition of judo athletes.

The “neural efficiency” hypothesis suggests that athletes with higher expertise may have higher cognitive ability with lower energetic demand. Interestingly, the fact that the expert group presented similar ERSP responses to perform the same cognitive task as the lower judo experienced group challenges this theory. In fact, Neubauer and Fink (2009) reviewed 54 studies related to the neural efficiency theory using a variety of cognitive tests and neuroimaging techniques (e.g., EEG, PET, and fMRI). They found that 53.7% of the studies confirmed the theory (i.e., negative correlation between performance and brain activity), while 29.6% of the studies found mixed results (i.e., partial support only for certain groups, under certain conditions/tasks or for certain brain areas), and the other 16.7% of the studies contradicted the theory (i.e., positive correlation between performance and brain activity). In addition, one would expect that lower amplitudes on ERP parameter may also be a marker of metabolic efficiency in athletes. However, different studies have shown that sports practice and regular practice in physical exercise programs may induce higher amplitudes and shorter latencies on N200 and P300 ERPs (Cheron et al., 2016). Our findings also showed similar patterns which BB had higher amplitude N200 in the precuneus and P300 in the middle PFC. Therefore, our findings together for ERSP and ERP does not support the neural efficiency theory and may suggest that sports expertise may induce an specific neural adaptation that may not necessarily be more efficient.

In the present study, the BB presented a 10-fold (or more) higher sport-specific experience compared to the WB group, which was reflected in higher aerobic fitness and judo-specific performance (see Table 1). Studies have consistently shown that cognitive performance, brain function, and structure is positively associated with aerobic fitness (Colcombe et al., 2006; Themanson and Hillman, 2006; Hillman et al., 2008; Chaddock et al., 2010b; Erickson et al., 2011). However, it is important to highlight that aerobic fitness specifically did not influence the results of the present study since statistical analysis included this parameter as a possible confounding factor. Also, educational history was not different between groups, which also may not have affected the results. This suggests that the different results in brain activity found here are likely related to sports experience.

Our limitations include the use of a cognitive task that represents only one aspect of the executive functions (Diamond, 2013), and also the absence of a sport-specific cognitive task or a cognitive task more associated with judo practice itself. We believe that this factor may help to explain why we found the behavioral performance to be similar between the expert and novice groups. On the EEG analysis, the reliance on source localization methods through algorithmic analysis presents some limitations, given that EEG does not provide the best spatial resolution. To consolidate the brain dynamics linked to the extensive practice of physical activity and/or regular sports practice, future studies should aim to use neuroimaging techniques with greater spatial resolution, such as functional magnetic resonance imaging.

The strengths of the present study include the assessment of brain electrical activity with a high-density EEG (i.e. 64 channels), which provides better coverage of the scalp and improved source localization; the analysis of source-based instead of a channel-based brain activity, which minimizes the problem related to the volume conduction effects (Denis et al., 2017). In addition, the use of ERSP along with ERP and source localization are interesting procedures that may provide a better understanding of brain function in response to long term sports training. Studies have indicated that neurocognitive functions are fundamental in the sports field (Nakata et al., 2010; Kim et al., 2014), while being able to provide a close management of the effects of the practice of physical exercise in the brain (Hillman et al., 2008). The findings of our study demonstrate that long-term training may modulate neural recruitment and the electrophysiological behavior of athletes with longer judo practice experience. Despite the similar behavioral response, judokas with higher expertise presented neural recruitment in cortical areas which are protagonists in the resolution of cognitive paradigms, as well as showing a greater N200 and P300 peak, and N450 area after receiving a visual/cognitive stimulus (Table 3).

## Conclusion

In conclusion, experienced judokas present no advantage for behavioral outcomes in selective attention tasks (i.e., RT, RTV, and accuracy) and frequencies bands (i.e. theta, alpha, and beta) as compared to a novice. Despite this, BB showed different pattern in allocating attentional resources in frontal area, greater conflict detection on parieto-occipital area, the lower speed in recruiting precuneus. Therefore, it seems that improved performance in individuals with higher sports expertise in judo may be limited to sports-specific stimulus and not to fundamental differences in the way these athletes selectively attend or inhibit irrelevant information on a context-free experimental cognitive task.

## Data Availability Statement

The datasets generated for this study are available on request to the corresponding author.

## Ethics Statement

Participants signed a written informed consent previous to their entrance in the study, following the Declaration of Helsinki. The study protocol was approved by the Institutional Ethics Committee (protocol number: 1.762.512).

## Author Contributions

HF and EF conceptualized this study. HF, DM, and PN contributed to the data collection. HF, DM, EF, and RM contributed to the data analysis. HF, DM, HB, HE, RM, and EF drafted the manuscript.

## Conflict of Interest

The authors declare that the research was conducted in the absence of any commercial or financial relationships that could be construed as a potential conflict of interest.
