# Supplementary figures and images for: Influence of Judo Experience on Neuroelectric Activity During a Selective Attention Task
Source: Front Psychol. 2020 Jan 9;10:2838. doi: 10.3389/fpsyg.2019.02838 (PMC6964796; doi:10.3389/fpsyg.2019.02838)

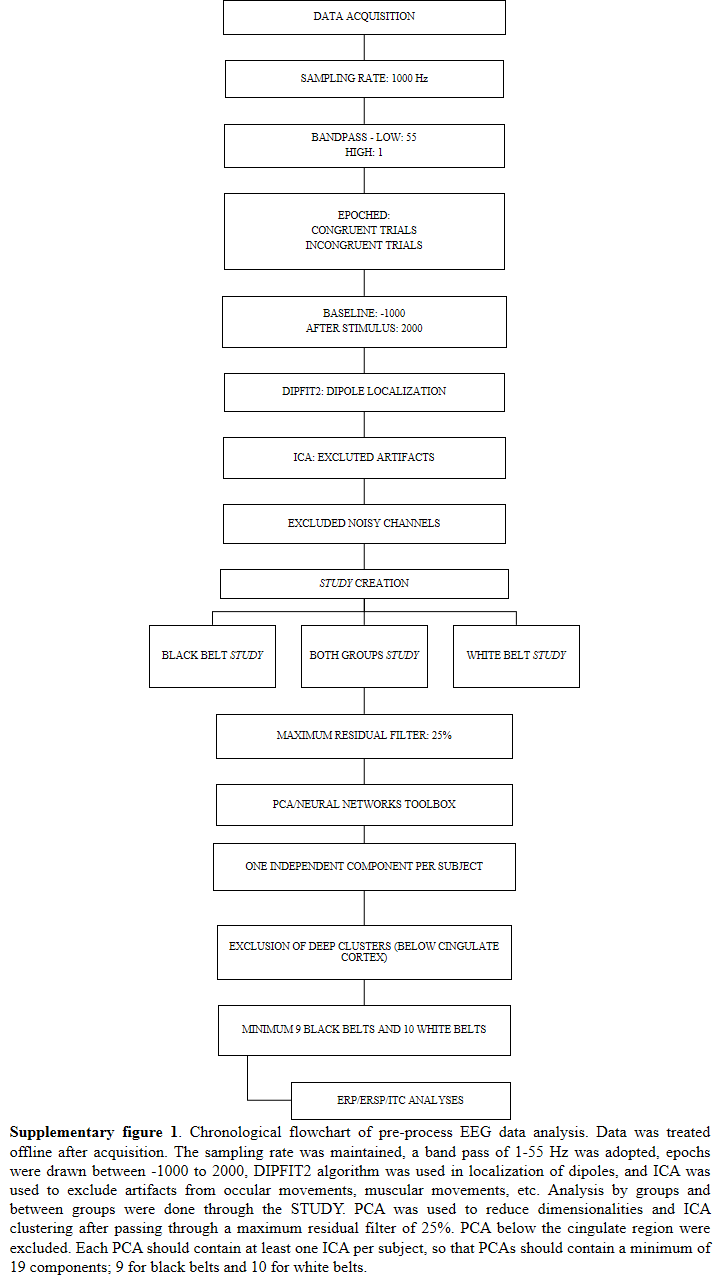

Supplement: Supplementary file 1 [file Image_1.tif]
