# Supplementary material for: Influence of Judo Experience on Neuroelectric Activity During a Selective Attention Task
Source: Front Psychol. 2020 Jan 9;10:2838. doi: 10.3389/fpsyg.2019.02838 (PMC6964796; doi:10.3389/fpsyg.2019.02838)
Supplement: Supplementary file 2 [file Table_1.docx]

**Supplementary Table 1.** General characteristics of experienced (n = 16) and novice (n = 18) judo athletes regarding body composition, aerobic fitness, and judo-specific fitness, after removing women from the sample (n = 2 and n = 3, respectively).

|  | | Black belt (n = 14) | White belt (n = 15) |  | F | df | t | *P* | Cohen’s d | Magnitude |
| --- | --- | --- | --- | --- | --- | --- | --- | --- | --- | --- |
| *Sociodemographics* | |  |  |  |  |  |  |  |  |  |
| Age (years) | | 27.1 ± 8.1 | 24.0 ± 5.1 |  | - | - | 1.220 | 0.233 | 0.45 | Small |
| Education (years) | | 19.7 ± 4.2 | 18.6 ± 3.0 |  | - | - | 0.688 | 0.499 | 0.30 | Small |
| *Body composition* |  | | | | | | | | |  |
| Height (cm) | | 172.0 ± 7.3 | 173.8 ± 7.3 |  | - | - | 0.660 | 0.515 | 0.24 | Small |
| Body mass (kg) | | 70.6 ± 11.4 | 70.5 ± 12.6 |  | - | - | 0.020 | 0.984 | 0.00 | Trivial |
| Percent of body fat (%) | | 17.6 ± 5.8 | 20.5 ± 8.8 |  | - | - | 0.964 | 0.345 | 0.38 | Small |
| Muscle mass (kg) | | 54.4 ± 8.7 | 49.9± 6.8 |  | - | - | 1.422 | 0.169 | 0.57 | Small |
| *Aerobic fitness test (maximal intermittent running test)* | | | | | | | | | |  |
| Distance covered (m) | | 931.6 ± 449.1* | 580.0 ± 177.5 |  | - | - | 2.614 | 0.016 | 1.02 | Moderate |
| Maximum running speed (km.h^-1^) | | 14.8 ± 0.81* | 14.2 ± 0.38 |  | - | - | 2.437 | 0.023 | 0.94 | Moderate |
| Maximum oxygen uptake (ml.kg^-1^.min^-1^) | | 44.22 ± 3.77* | 41.0 ± 1.68 |  | - | - | 2.745 | 0.012 | 1.10 | Moderate |
| *Special Judo Fitness Test (SJFT)* | | | | | | | | | |  |
| Number of throws in the SJFT | | 25.28 ± 2.16* | 21.72 ± 2.49 |  | - | - | 3.818 | 0.001 | 1.52 | Large |
| Final heart rate (bpm) | | 178.2 ± 10.65 | 180.9 ± 13.42 |  | - | - | 0.545 | 0.591 | 0.22 | Small |
| Heart rate after 1 minute (bpm) | | 154.78 ± 12.65 | 155.81 ± 20.74 |  | - | - | 0.154 | 0.879 | 0.05 | Trivial |
| SJFT index (a.u.) | | 13.26 ± 1.27* | 15.54 ± 1.95 |  | - | - | 3.381 | 0.003 | 1.38 | Large |
| *Stroop Matching Test* | |  |  |  |  |  |  |  |  |  |
| Congruent accuracy (%) | | 99.5 ± 1.0 | 97.0 ± 8.1 |  | 3.245 | 1,27 | - | 0.083 | 0.43 | Small |
| Incongruent accuracy (%) | | 93.7 ± 9.1 | 96.5 ± 3.6 |  | 3.245 | 1,27 | - | 0.083 | 0.40 | Small |
| Congruent response time (ms) | | 676.0 ± 110.9 | 652.6 ± 97.84 |  | 0.541 | 1,27 | - | 0.468 | 0.22 | Small |
| Incongruent response time (ms) | | 813.2 ± 118.8 | 801.7 ± 109.5 |  | 0.541 | 1,27 | - | 0.468 | 0.10 | Trivial |
| Congruent response time variability (ms) | | 120.1 ± 36.0 | 124.22 ± 38.8 |  | 0.034 | 1,27 | - | 0.854 | 0.10 | Trivial |
| Incongruent response time variability (ms) | | 146.0 ± 30.7 | 147.1 ± 28.4 |  | 0.034 | 1,27 | - | 0.854 | 0.03 | Trivial |
| Stroop effect (ms) | | 137.1 ± 27.2 | 149.2 ± 54.6 |  | - | - | 0.743 | 0.464 | 0.28 | Small |
