# Supplementary material for: Influence of Judo Experience on Neuroelectric Activity During a Selective Attention Task
Source: Front Psychol. 2020 Jan 9;10:2838. doi: 10.3389/fpsyg.2019.02838 (PMC6964796; doi:10.3389/fpsyg.2019.02838)
Supplement: Supplementary file 3 [file Table_2.docx]

**Supplementary Table 2**. Descriptive statistics and correlations among Special Judo Fitness Test measures and Yoyo Intermittent Recovery Test 1 measures in novice (white belt; n = 18) and experienced (black belt; n = 16) judo athletes (overall n = 34).

| Measure | Mean | 95%CI | Min | Max | 1 | 2 | 3 | 4 | 5 | 6 | 7 |
| --- | --- | --- | --- | --- | --- | --- | --- | --- | --- | --- | --- |
| 1 - Number of throws in the SJFT (n) | 29 | 17.7 - 29.9 | 19 | 29 |  | .03 | .15 | -.83** | .29 | .27 | .29 |
| 2 - Final heart rate (bpm) | 203 | 154.6 - 203.5 | 161 | 203 |  |  | .68** | .44* | .11 | .10 | .11 |
| 3 - Heart rate after 1 minute (bpm) | 188 | 121.8 - 189.1 | 127 | 188 |  |  |  | .38 | -.04 | -.05 | -.04 |
| 4 - SJFT index (a.u.) | 14.18 | 10.1 - 18.2 | 11.48 | 18.73 |  |  |  |  | -.23 | -.23 | -.23 |
| 5 - Distance covered (m) | 682 | 105.0 - 1469.0 | 200 | 1820 |  |  |  |  |  | .95** | 1** |
| 6 - Maximum running speed (km.h^-1^) | 14.45 | 13.0 - 15.8 | 13.5 | 16.5 |  |  |  |  |  |  | .95** |
| 7 - VO_2max_ (ml.kg^-1^.min^-1^) | 42.12 | 35.5 - 48.7 | 38.08 | 51.69 |  |  |  |  |  |  |  |

Note: SJFT = Special judo fitness test; 95%CI = 95% confidence intervals; * and ** statiscally significant (P<0.05 and P<0.001, respectively).
