# Supplementary material for: Influence of Judo Experience on Neuroelectric Activity During a Selective Attention Task
Source: Front Psychol. 2020 Jan 9;10:2838. doi: 10.3389/fpsyg.2019.02838 (PMC6964796; doi:10.3389/fpsyg.2019.02838)
Supplement: Supplementary file 4 [file Table_3.docx]

**Supplementary Table 3.** Neuroanatomical areas, number of components, Brodmann areas and Talairach coordinates of centroids for experienced and novice judo athletes.

| **Black Belt (n = 16)** | | | | | |  | **White Belt (n = 18)** | | | | | |
| --- | --- | --- | --- | --- | --- | --- | --- | --- | --- | --- | --- | --- |
| **Lobe / Areas** | **NC** | **BA** | **Talairach coordinates** | | |  | **Lobe / Areas** | **NC** | **BA** | **Talairach coordinates** | | |
|  |  |  | **X** | **Y** | **Z** |  |  |  |  | **X** | **Y** | **X** |
| Frontal Lobe / middle frontal gyrus (L) | 9 | 10 | -35 | 52 | 6 |  | Frontal Lobe / middle frontal gyrus (L) | 11 | 10 | -28 | 44 | 17 |
| Frontal Lobe / superior frontal gyrus (L) | 10 | 9 | -14 | 50 | 31 |  | Frontal Lobe / precentral gyrus (L) | 10 | 4 | -37 | -18 | 56 |
| Frontal Lobe / medial frontal gyrus (L) | 9 | 11 | -7 | 55 | -13 |  | Frontal Lobe / precentral gyrus (R) | 12 | 4 | 16 | -25 | 65 |
| Limbic Lobe / cingulate gyrus (L) | 10 | 24 | -19 | -2 | 49 |  | Parietal Lobe / precuneus (R) | 11 | 39 | 39 | -66 | 33 |
| Occipital Lobe / cuneus (R) | 11 | 7 | 14 | -74 | 31 |  | Occipital Lobe / cuneus (L) | 12 | 19 | -3 | -85 | 30 |
| Parietal Lobe / precuneus (L) | 13 | 7 | -18 | -59 | 37 |  | Parietal Lobe / precuneus (L) | 14 | 31 | -12 | -49 | 26 |

**Note:** NC = number of components; BA = Brodmann area; L = left; R = right.
