# Supplementary material for: Influence of Judo Experience on Neuroelectric Activity During a Selective Attention Task
Source: Front Psychol. 2020 Jan 9;10:2838. doi: 10.3389/fpsyg.2019.02838 (PMC6964796; doi:10.3389/fpsyg.2019.02838)
Supplement: Supplementary file 5 [file Table_4.docx]

**Supplementary Table 4**. Areas that presented more components clustered during cognitive test response in black (n = 16) and white (n = 18) belts judo athletes (overall n = 34).

| **Lobe / Areas** | **Number of components** | **Brodmann area** |  | **Talairach coordinates** |  |
| --- | --- | --- | --- | --- | --- |
|  |  |  | **X** | **Y** | **Z** |
| Frontal Lobe / middle frontal gyrus | 22 | 10 | -33 | 41 | 19 |
| Occipital Lobe / Cuneus | 24 | 19 | 3 | -81 | 36 |
| Parietal Lobe / Precuneus | 25 | 31 | -15 | -49 | 32 |
